# Editorial: Reviews in sport and exercise nutrition

**DOI:** 10.3389/fspor.2024.1365741

**Published:** 2024-01-25

**Authors:** David C. Nieman

**Affiliations:** Human Performance Laboratory, Department of Biology, North Carolina Research Campus, Appalachian State University, Boone, NC, United States

**Keywords:** sports nutrition, personalized nutrition, vitamin D, microbiome, body composition

**Editorial on the Research Topic** Reviews in sport and exercise nutrition

This research topic highlighted scholarly review papers that explored key topics in sport and exercise nutrition (SEN). The four papers in this research topic included the application of evidence-based guidelines for sport and exercise nutrition practitioners, a meta-analysis on the influence of vitamin D supplements on muscle strength and power, a narrative review on the effect of dietary and physical activity patterns on the aging gut microbiome, and a review of the influence of body composition on performance and health among military personnel.

Sport and exercise nutrition practitioners are faced with the daunting challenge of communicating recent scientific findings into practical recommendations and guidelines for athletes in a variety of sports (1, 2). In the article, *Bridging the gap: Evidence-based practice guidelines for sports nutritionists*, Ritson et al. provided an adapted version of evidence-based guidelines for SEN professionals. The authors proposed this evidence-based practice (EBP) definition for SEN: “A thorough, integrated approach to the nutritional support of athletes based on the best available evidence and expert professional judgment, taking into account the athlete's key performance determinants, values, goals, personal preferences and circumstances, as well as the practitioner's work context.” A five-step adapted EBP process was described as follows: (1) ask pertinent questions, (2) acquire the best available evidence, (3) appraise the evidence critically, (4) apply and integrate the evidence appropriately, and (5) audit these processes for continued improvement. The emphasis was placed on integrating the EBP process with professional experience and the specific needs and values of each individual athlete.

Vitamin D is involved in multiple physiological processes, including bone development and maintenance, immune function, and skeletal muscle function. Some athletes are vitamin D deficient, but the extent remains a subject of discussion due to the inconsistent screening guidelines. The use of vitamin D supplementation to improve muscle function is popular among athletes, but previous systematic reviews of the published literature on this topic have been inconsistent (3–6). In the article, *Effects of vitamin D supplementation on maximal strength and power in athletes: a systematic review and meta-analysis of randomized controlled trials*, Sist et al. quantitatively summarized the current literature and selected 12 randomized controlled trials (RCTs) that used standard measurements for maximal strength (1 RM test for multi-joint exercises) and power (vertical jump). The findings of this meta-analysis indicated that there was no effect of vitamin D supplementation on upper and lower body muscle strength despite meaningful increases in 25-hydroxyvitamin D [25(OH)D]. The authors emphasized the necessity for additional studies with larger athletic populations, longer supplementation periods, and improved assessments of training protocols. Well-designed studies that control for both sun exposure and dietary intake on vitamin D insufficient or deficient athletes are lacking.

The gut microbiome has an intimate influence on human health and can be modified by lifestyle factors (7, 8). In the narrative review paper, *Growing old together: What we know about the influence of diet and exercise on the aging host’s gut microbiome*, Brooks et al. summarized published information on how the gut microbiome changes with age and how this may be altered by dietary and physical activity patterns. The authors emphasized that the interplay between aging, diet, exercise, and the gut microbiome is an emerging area of scientific endeavor and that scant data exist to draw definitive conclusions. Evidence was reviewed that a diverse microbiome is promoted through a plant-based dietary pattern and facilitates increased colon metabolite production, improved gut mucosal barrier function, and enhanced immunity. The effects of increased physical activity patterns on microbiome diversity are not as well established.

Body composition is an important determinant of both health and performance in all humans including active-duty military personnel (9). In the paper, *Body composition as a marker of performance and health in military personnel*, Cialdella-Kam et al. reviewed articles from the past 20 years on body composition and associations to performance, health, and disordered eating behaviors in military personnel. The linkage between body composition and military performance and preparedness is poorly understood, especially among female warfighters in the Air Force and Navy. Limited data support that fat-free mass is a better determinant of performance than percent body fat and that adiposity is related to higher inflammation, decreased cognitive health, and increased risk for musculoskeletal injuries in the military population. The authors urged that a comprehensive survey on weight cycling, disordered eating, and weight management would be valuable to determine the prevalence and extent of these issues. This information, along with performance data, would provide guidance on the relevance of existing and varied body composition standards in the military branches.
